# Contralateral neurological symptoms after ACDF for radiculopathic cervical spondylosis:A case report

**DOI:** 10.1016/j.ijscr.2025.111634

**Published:** 2025-07-09

**Authors:** Ruoheng Yin, Weiyu Jiang, Weihu Ma, Yunlin Chen

**Affiliations:** a Ningbo University Health Science Center, Ningbo, Zhejiang, China; b Spine Surgure Center, Ningbo No.6 Hospital, Ningbo, Zhejiang, China; c Ningbo Clinical Research Center for Orthopedics, Sports Medicine & Rehabilitation, Ningbo, Zhejiang, China

**Keywords:** Radiculopathic cervical spondylosis, Neurological symptoms, Revision surgery, ACDF, Case report

## Abstract

**Introduction:**

Anterior cervical discectomy and fusion (ACDF) is the standard surgical procedure for cervical radiculopathy. However ACDF has certain limitations, a small number of patients may require a second surgery after ACDF, we further analyzed and summarized our experience regarding curvature restoration and management of the asymptomatic side in cervical radiculopathy.

**Presentation of case:**

A 67-year-old male patient experienced neck and left arm pain for over a decade, worsening recently. Following admission, the patient underwent an anterior cervical discectomy and fusion (ACDF) procedure. Postoperatively, the patient began to exhibit symptoms on the opposite (right) side. Follow-up imaging showed a reduction in disc space height at the posterior aspect of the C5/6 level, along with a slight posterior shift of the C5 vertebra. A revision surgery was performed. The symptoms were alleviated.

**Discussion:**

For patients with cervical foraminal stenosis, restoring posterior disc height is critical for enlarging the foramen. In contrast, increasing the lordotic angle of the fused segment does not significantly improve foraminal dimensions.

**Conclusion:**

For patients suffering from radiculopathy caused by foraminal stenosis, we suggest the following guidelines:1) It is not necessary to excessively focus on the restoration of cervical lordosis，maintaining the height and width of the intervertebral foramen is more critical. 2)When distracting the intervertebral space, placing the cage in a relatively posterior position helps to preserve the height of the posterior disc space 0.3)In the short term, stable vertebral slippage without nerve compression does not necessitate inclusion in the fusion construct.

## Highlights

• Although indirect decompression on the asymptomatic side has demonstrated favorable therapeutic outcomes in previous surgical cases, this particular patient exhibited postoperative symptom exacerbation on the initially asymptomatic side.
• For patients with cervical foraminal stenosis, restoring posterior disc height is critical for enlarging the foramen. In contrast, increasing the lordotic angle of the fused segment does not significantly improve foraminal dimensions.
• It is not necessary to excessively focus on the restoration of cervical lordosis，maintaining the height and width of the intervertebral foramen is more critical.
• When distracting the intervertebral space, placing the cage in a relatively posterior position helps to preserve the height of the posterior disc space.
• In the short term, stable vertebral slippage without nerve compression does not necessitate inclusion in the fusion construct.

## 1 Introduction

Radiculopathic cervical spondylosis refers to age-related degenerative changes in the cervical spine, such as intervertebral disc herniation, hypertrophy of the facet and uncovertebral joints, these conditions interact, causing soft or hard foraminal stenosis, which compresses the nerve roots, results in clinical symptoms like neck pain and radiating pain in the upper limbs [1,2].Anterior cervical discectomy and fusion (ACDF) can reconstruct the normal physiological curvature of the cervical spine, effectively restore disc space height, and directly or indirectly enlarge the neural foramina, thereby relieving nerve compression. This procedure is known for its strong stability, minimal invasiveness, rapid recovery, and definitive therapeutic effects, making it the standard surgical approach for treating radiculopathic cervical spondylosis. Many studies have confirmed its efficacy in managing cervical spondylosis [3].However, ACDF has certain limitations, such as a limited surgical field of view and difficulty in completely removing ossification of the posterior longitudinal ligament and the foramen [4]. Consequently, a small number of patients may require a second surgery after ACDF. In the case report below, we describe a 67-year-old patient with radiculopathic cervical spondylosis who developed contralateral nerve root symptoms after ACDF and subsequently required revision surgery. While previous studies have demonstrated favorable outcomes of indirect decompression on the asymptomatic side, but the present case exhibited postoperative symptom exacerbation on this side. Based on this finding, we further analyzed and summarized our experience regarding curvature restoration and management of the asymptomatic side in cervical radiculopathy. This case report has been reported in line with the SCARE checklist [5].

### 1.1 Case presentation

The patient is a 67-year-old male who has experienced recurrent neck and left upper limb pain for over 10 years, with symptoms worsening over the past 20 days.

He reported pain and numbness along the radial side of the left arm and forearm, radiating to the thumb and index finger. Physical examination showed a positive Eation test, with grade 5 muscle strength and grip strength in the left upper limb. Physiological reflexes were active in all four limbs, and no pathological reflexes were observed. His VAS pain score was 8–9.

**Fig. 1:**
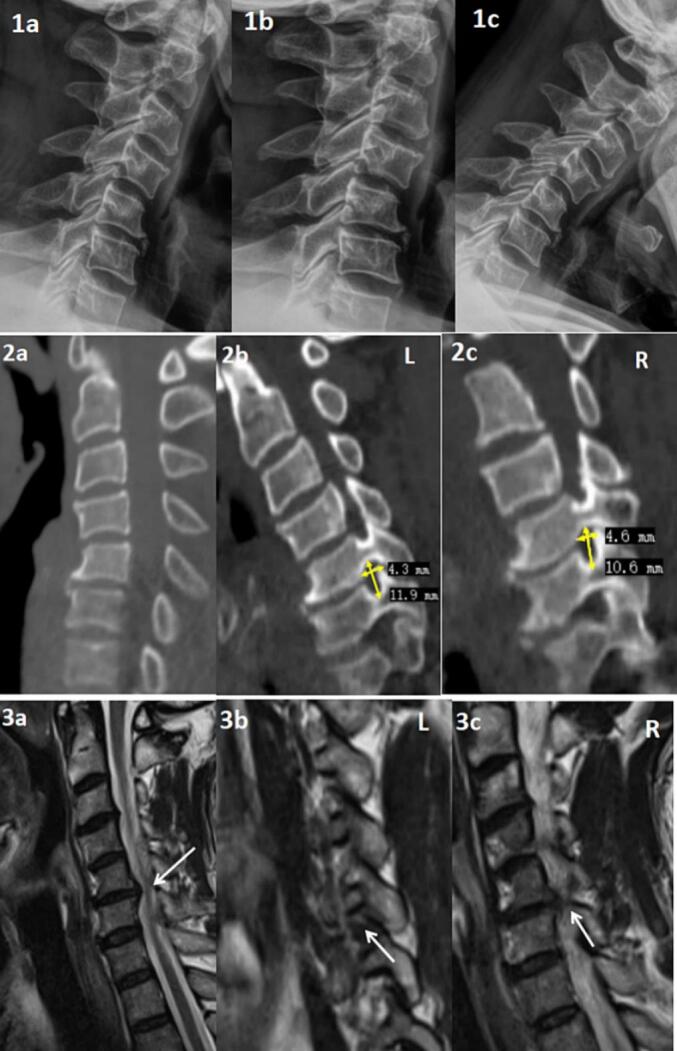
Preoperative Imaging (1a-c) Preoperative X-rays:(1a) Anteroposterior view(1*b*)Extension view(1*c*)Flexion view.(2a-c)Preoperative CT:(2a)cervical kyphosis.(2b) stenosis of the left foramen with a height of approximately 11.3 mm and a width of 4.3 mm.(2c)stenosis of the right foramen with a height of approximately 10.9 mm and a width of 4.6 mm.(3a-c)Preoperative MRI:(3a)White arrows indicate mild spinal cord compression at the C5/6 level.(3b) White arrows indicate stenosis of the left foramen at the C5/6 level.(3c) White arrows indicate stenosis of the right foramen at the C5/6 level.

Preoperative X-rays showed mild kyphosis of the cervical spine **(**Fig. 1**.1a)**, and flexion-extension views demonstrated instability in the alignment of the C4-C6 vertebrae **(1b, 1c)**. CT scans revealed cervical kyphosis**(2a)** with bilateral foraminal stenosis at the C5/6 level. Measurements indicated a left foramen height of approximately 11.3 mm and width of 4.3 mm **(2b)**, and a right foramen height of about 10.9 mm and width of 4.6 mm **(2c)**. MRI showed straightening of the cervical lordosis with disc herniation at the C5/6 level, causing mild spinal cord compression**(3a)**, though the patient did not exhibit significant myelopathic symptoms. Bilateral foraminal stenosis with nerve root compression was noted at the C5/6 level**(3b, c)**, with more pronounced symptoms in the left upper limb and no radicular symptoms on the right side.

Based on the patient's symptoms and imaging findings, the bony structure was relatively well-preserved, with lateral disc protrusion and hypertrophy of the ligament flava causing soft foraminal stenosis, primarily responsible for the radicular symptoms on the left side. We decided to perform an ACDF procedure under microscopy. A conventional transverse incision was made medial to the sternocleidomastoid muscle, and the layers were dissected to expose the C5/6 level. Under microscopic guidance, the C5/6 disc space was distracted, and focused decompression was performed on the left side. The C5/6 disc, hypertrophic soft tissues, and part of the posterior longitudinal ligament were removed. Since there were no radicular symptoms on the right side, indirect decompression was achieved through distraction of the disc space alone. A 6 mm interbody fusion cage was then placed at the anterior edge of the C5/6 disc space.

**Fig. 2:**
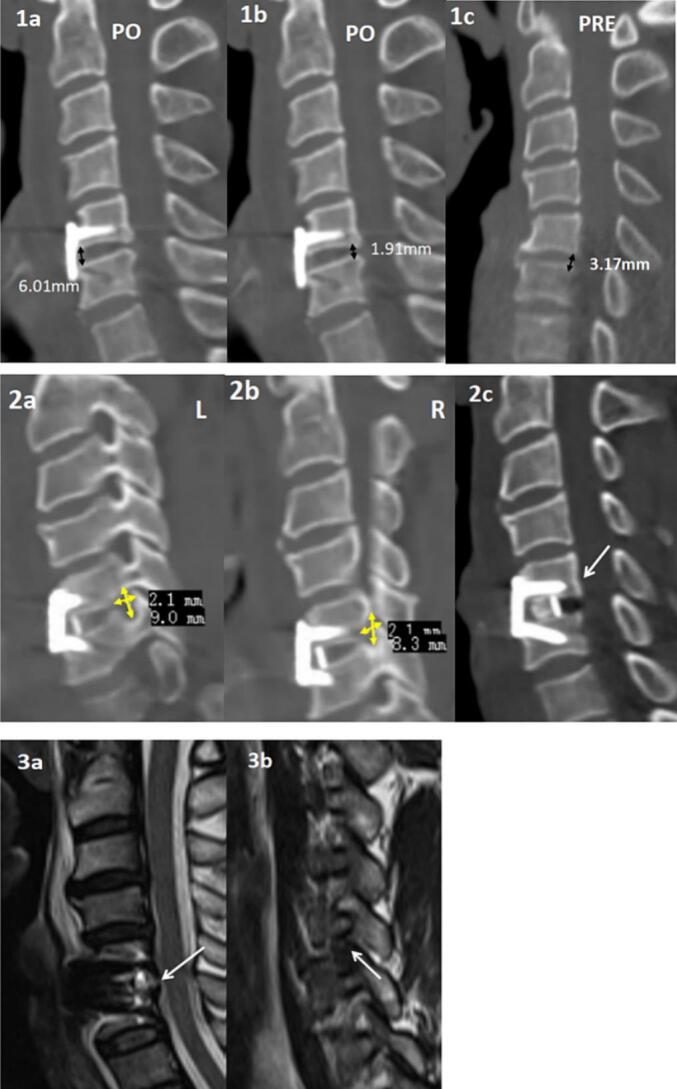
(1a-2c) Postoperative CT:(1a) Anterior disc space height postoperatively(1b) Posterior disc space height postoperatively(1c) Preoperative posterior disc space height(2a) Postoperative left foramen height and width(2b) Postoperative right foramen height and width(2c) White arrow indicating mild posterior displacement of C5 vertebra(3a-b) Postoperative MRI:(3a) White arrow indicating thorough decompression of the spinal cord(3b) White arrow indicating stenosis of the right foramen.

However, postoperatively, the patient reported significant improvement in left-sided pain but developed new symptoms of increased pain in the right upper arm, accompanied by pain and numbness in the dorsal aspect of the right hand and the interosseous muscles. The left VAS pain score was 3, while the right side was 8. Follow-up CT scans showed good positioning of the anterior plate and screws at C5/6.The anterior disc space at the surgical level was adequately distracted **(**Fig. 2**.1a)**, but the posterior disc space height on the right side measured only 1.9 mm **(2.1b)**, compared to 3.2 mm preoperatively **(2.1c)**, indicating a significant loss of posterior disc space height. The left foramen measured approximately 9.0 mm in height and 2.1 mm in width **(2a)**, while the right foramen measured about 8.3 mm in height and 2.1 mm in width **(2b).** Both foramina were further narrowed compared to preoperative CT, and a slight posterior displacement of the C5 vertebra was visible on the sagittal view **(2c)**. Postoperative MRI confirmed the proper positioning of the pedicle screws, plate, and cage at C5/6, with heterogeneous disc space signals and edema in the anterior cervical soft tissues **(3a)**. The right foramen was further narrowed **(3b).**

After the ACDF surgery, the patient experienced further narrowing of the bilateral foramina and the development of radiculopathic symptoms on the contralateral side. Despite conservative treatment, the symptoms persisted, and the patient, after being fully informed, opted for revision surgery. The original incision was used to access the C5/6 disc space, and the layers were dissected to expose the area. The original plate, screws, and cage were removed, revealing complete decompression on the left side. The disc space was distracted, and a gun clamp was used to resect part of the right uncovertebral joint, decompressing the nerve root. The C5 vertebra was then realigned anteriorly to restore the height and width of the foramen. A 7 mm interbody fusion cage was inserted, further distracting the C5/6 disc space, with the cage positioned slightly more posteriorly than in the initial surgery.

Postoperatively, the patient reported significant relief from pain and numbness in both upper limbs. CT scans confirmed proper positioning of the screws and fusion cage, with a posterior disc space height of 6.1 mm (Fig. 3.1a). The left foramen measured 12.2 mm (height) × 4.0 mm (width) (Fig. 2a), and the right foramen measured 11.3 mm (height) × 4.9 mm (width) (Fig. 2b). At the one-month follow-up, the patient demonstrated marked improvement in pain and numbness compared to preperative symptoms.

**Fig. 3:**
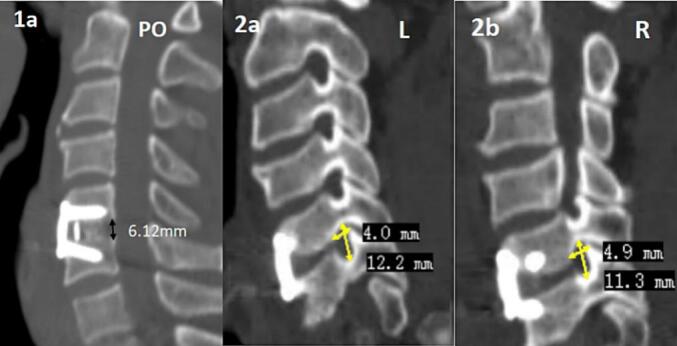
Post-revision Surgery CT:(1a) Post-revision posterior disc space height(2a) Post-revision left foramen height and width(2b) Post-revision right foramen height and width.

## 2 Discussion

While ACDF is widely regarded as an effective treatment, it has inherent limitations, including a restricted surgical field of view and challenges in fully addressing ossification of the posterior longitudinal ligament and foraminal stenosis [4].Previous studies indicate that the primary reasons for reoperation after ACDF include pain recurrence, implant collapse, and pseudarthrosis, typically occurring more than six months postoperatively [6,7]. Early reoperations before discharge are often due to postoperative hematoma, with an incidence rate of approximately 0.40 %, associated with factors such as multi-level surgery, elevated preoperative INR, low BMI, high ASA score, preoperative anemia, and male gender [8].In this case, the patient experienced a worsening of symptoms just one day after ACDF. Based on the imaging results, we concluded that the process of restoring the anterior convexity may have triggered a posterior vertebral slippage and a reduction in the posterior disc space, ultimately has led to a further decrease in the size of the intervertebral foramen. Therefore, the patient opted for a revision surgery. Although indirect decompression on the asymptomatic side has demonstrated favorable therapeutic outcomes in previous surgical cases, this particular patient exhibited postoperative symptom exacerbation on the initially asymptomatic side. We suggest that the optimal balance between improving radicular symptoms and restoring cervical spine curvature warrants further discussion.

Previous research has shown that the average width and height of the neural foramen are approximately 6–7 mm and 8–11 mm, respectively [9]. If the foramen width is 4.35 mm or less, the likelihood of persistent pain increases [10]. Restoring the disc space height is beneficial for enlarging the foramen, but excessive distraction can increase mechanical stress on adjacent segments [11]. Therefore, achieving an appropriate disc space height is crucial for improving symptoms after ACDF.A study by Yang et al. [12]found that excessive distraction may only expand the anterior column without affecting the posterior column, leading to non-parallel endplates and a reduction in posterior disc space height. They suggested that the optimal distraction should be 160 % of the average height of the disc spaces.

Cervical sagittal imbalance increases energy expenditure to maintain balance and mobility, predisposing patients to cervical disc degeneration [13]. in this case, the patient presented with preoperative cervical straightening. Our surgical goal was to relieving neural compression while restore cervical lordosis to improve sagittal alignment. To achieve this, the cage was positioned at the more anterior edge of the disc space, which may have contributed to the loss of posterior disc height at C5/6.During the initial surgery, while adequate decompression was performed for the symptomatic left nerve root, the right side was only indirectly decompressed through disc space distraction. The resultant loss of posterior disc height further narrowed the right foramen, leading to contralateral radiculopathic symptoms. For patients with cervical foraminal stenosis, restoring posterior disc height is critical for enlarging the foramen. In contrast, increasing the lordotic angle of the fused segment does not significantly improve foraminal dimensions. Research by Suk [14] demonstrated that posterior disc height is strongly associated with postoperative foraminal size. Increased foraminal size correlates with reduced postoperative arm pain, while graft positioning negatively affects posterior disc height. Additionally, an increased lordotic angle in the fused segment is inversely related to foraminal size. Therefore, positioning the cage more posteriorly is advantageous for maintaining posterior disc height and enlarging the foramen.

In this case, a mild posterior slippage of the C5 vertebra may have contributed to the development of contralateral radiculopathy postoperatively, potentially linked to the inherent instability of the C4-C6 vertebral alignment. Vertebral slippage is a known cause of cervical radiculopathy, and the management of adjacent segment instability—whether preoperative or postoperative—has been a topic of debate. Segar [15] compared outcomes of ACDF in 264 patients, categorizing them into those with and without adjacent segment disease (ASD). Both groups demonstrated significant improvement from baseline, with no notable differences in outcomes, suggesting that ASD is not a predictor of postoperative results. Similar results were reported by Goyal [16], who noted comparable improvements in all measures of bodily pain and functional outcomes between the two groups. Therefore, in the short term, stable vertebral slippage without nerve compression does not necessitate inclusion in the fusion construct.

## 3 Conclusion

For patients suffering from radiculopathy caused by foraminal stenosis, we suggest the following guidelines:1) It is not necessary to excessively focus on the restoration of cervical lordosis, maintaining the height and width of the intervertebral foramen is more critical. 2)When distracting the intervertebral space, placing the cage in a relatively posterior position helps to preserve the height of the posterior disc space 0.3)For patients with unstable vertebral alignment, it is recommended to minimize the risk of posterior vertebral slippage during the process of restoring lordosis, in the short term, stable vertebral slippage without nerve compression does not necessitate inclusion in the fusion construct.

## Consent for publication

The patient has signed written informed consent for clinical details and imaging data to be published.

## Consent to participate

Consent for publication was obtained from publication.

### Ethics approval and consent to participate

This study had been approved by Ethics Committee of The NINGBO NO.6 hospital. Written informed consent was obtained from the patient for publication of this case report and accompanying images.

## Ethics approval

This study had been approved by Ethics Committee of hospital. Written informed consent was obtained from the patient for publication of this case report and accompanying images.

## Funding

This work was supported by Medical and Health Science and Technology Project of Zhejiang Province (Grant numbers 2024KY381) and Ningbo Clinical Research Center for Orthopedics, Sports Medicine & Rehabilitation (2024L004).

## Authors' contributions

All authors participated in the surgical procedures and patient management. All authors contributed to the study conception and design. Ruoheng Yin design of the study and drafted the manuscript. Chen and Ma were responsible for the data collection and measurement of radiographic data. Weiyu Jiang performed revised the manuscript critically and gave final approval of the version to be published. All authors read and approved the final manuscript.

## Guarantor

Weiyu Jiang.

## Declaration of competing interest

The authors have no relevant financial or non-financial interests.

## Data Availability

The data and materials that support the findings of this study are available from the corresponding author upon reasonable request.
